# Insufficiently Defined Genetic Background Confounds Phenotypes in Transgenic Studies As Exemplified by Malaria Infection in Tlr9 Knockout Mice

**DOI:** 10.1371/journal.pone.0027131

**Published:** 2011-11-11

**Authors:** Nathalie Geurts, Erik Martens, Sebastien Verhenne, Natacha Lays, Greet Thijs, Stefan Magez, Bénédicte Cauwe, Sandra Li, Hubertine Heremans, Ghislain Opdenakker, Philippe E. Van den Steen

**Affiliations:** 1 Laboratory of Immunobiology, Rega Institute for Medical Research, University of Leuven, Leuven, Belgium; 2 Laboratory for Cellular and Molecular Immunology, Vrije Universiteit Brussel, Brussels, Belgium; Agency for Science, Technology and Research - Singapore Immunology Network, Singapore

## Abstract

The use of genetically modified mice, i.e. transgenic as well as gene knockout (KO) and knock-in mice, has become an established tool to study gene function in many animal models for human diseases . However, a gene functions in a particular genomic context. This implies the importance of a well-defined homogenous genetic background for the analysis and interpretation of phenotypes associated with genetic mutations. By studying a *Plasmodium chabaudi chabaudi* AS (*Pc*AS) malaria infection in mice bearing a *TLR9* null mutation, we found an increased susceptibility to infection, i.e. higher parasitemia levels and increased mortality. However, this was not triggered by the deficient TLR9 gene itself. Instead, this disease phenotype was dependent on the heterogeneous genetic background of the mice, which appeared insufficiently defined as determined by single nucleotide polymorphism (SNP) analysis. Hence, it is of critical importance to study gene KO phenotypes on a homogenous genetic background identical to that of their wild type (WT) control counterparts. In particular, to avoid problems related to an insufficiently defined genetic background, we advocate that for each study involving genetically modified mice, at least a detailed description of the origin and genetic background of both the WT control and the altered strain of mice is essential.

## Introduction

The major histocompatibility complex (MHC) on human chromosome 6 covers a large genomic region with a central role in determining the degree of disease susceptibility. In this locus, small gene polymorphisms, typically located in alleles in charge of protein processing and presentation as well as immune regulation, are associated with several human autoimmune diseases, e.g. multiple sclerosis, type 1 diabetes and rheumatoid arthritis, but also with infectious diseases, e.g. malaria [1]–[6]. Aside the MHC having a major impact on immune phenotypes, many genes outside this susceptibility locus contribute to immunomodulation. This is increasingly recognized by genome-wide association studies (GWAS) in which genes encoding cytokines, cytokine receptors and pattern recognition receptors (PRRs, e.g. Toll-like receptors (TLRs), RIG-I-like receptors (RLRs), Nod-like receptors (NLRs)) are identified to play small but definite effects that may synergize [7], [8]. In addition, single nucleotide polymorphisms (SNPs) in TLRs are found to correlate with susceptibility to infectious diseases [9]. For instance, polymorphisms in *TLR1*, *TLR4* and *TLR9* are associated with an aggravated clinical status of malaria during pregnancy [10], [11]. Polymorphisms in *TLR4* are also reported to play a role in controlling the parasitemia level in malaria [12]. Furthermore, in malaria, alleles associated with sickle-cell anemia, thalassemias, and glucose-6-phosphate dehydrogenase deficiency confer protective effects, whereas polymorphisms in the *TNF-α* gene have been linked to increased risk of cerebral malaria (CM) [13]. Hence, complex host genetics, in addition to environmental factors, are invoked in defining susceptibility or resistance against several diseases.

In malaria research, many groups attempted to examine the role of TLRs by using TLR^−/−^ mice. Both in human and experimental malaria, enhanced TLR activation is suggested to prime proinflammatory cytokine responses (IL-12, IFN-γ, TNF-α), which in turn can favor host hyperresponsiveness to TLR agonists during acute malaria. Subsequently, excessive inflammation might contribute to malaria pathology, such as fever, CM and anemia [14], [15]. Therapeutical treatment with TLR antagonists was found to diminish TLR activation and to prevent the development of experimental cerebral malaria (ECM) [16]. Tolerance to TLR signaling, however, was observed in a murine malaria model at later stages of infection and was paralleled by an anti-inflammatory cytokine response [17]. This biphasic modulation of the immune system might reflect a mechanism to balance pro- and anti-inflammatory responses to avoid severe pathology.

The fact that the phenotype of a single gene mutation is frequently modulated by a large number of background genes has been illustrated by Griffith *et al.*, who have studied the role of TLR signaling in the development of ECM [18]. It was shown that knocking out MyD88 or TLR9 in the CM-susceptible Th_1_-permissive C57Bl/6 background turns out into a resistant phenotype, whereas in CM-resistant Th_2_-permissive Balb/c mice, interruption of the TLR pathway by deleting MyD88 results in development of CM and increased mortality. Thus, two congenic strains carrying the same null mutation can have a divergent phenotype dependent on their genetic background. This is, however, not unique to malaria-related phenotypes [19]–[21]. Coban *et al.* also revealed that TLR2-, TLR9- and MyD88-dependent signaling is essential in murine CM on a C57Bl/6 background [22]. However, controversies on the contribution of the TLR cascade to the development of experimental CM pathogenesis exist as several studies ascertain that murine CM is independent of TLR signaling [23], [24]. In addition, the route of infection also appears to influence the effect of the TLR pathway on CM [25].

*Plasmodium spp.* have been shown to contain ligands for TLRs. Besides TLR2 that recognizes malarial glycosylphosphatidylinositol [26], TLR9 gained attention since hemozoin or malaria pigment, which is a crystalline detoxification product of heme formed in the food vacuole of parasites during hemoglobin digestion, has been described as a ligand of TLR9 able to induce immune activation [27], [28]. However, it is still a matter of debate whether hemozoin itself binds this receptor, whether malaria pigment carries plasmodial DNA to TLR9 or whether the TLR9 ligand is a histone-DNA complex [29]–[32]. Recently, it was discovered that several hemozoin effects are dependent on its stable interaction with host fibrinogen and the activation of receptors TLR4/integrin by this complex [33].

In this study, we attempted to further examine the role of TLR9 in a *Plasmodium chabaudi chabaudi* (*Pc*AS) infection model. First, we used a common practice strategy, by studying and comparing commercial wild type mice from Janvier (WT(Jv)) with the TLR9^−/−^ mice that we originally received (OR). Subsequently, the validity of this strategy was investigated by performing an additional backcross and by in-depth investigation of the genetic background of these mice. Our data demonstrate that phenotypic differences observed between mutant and control mice can simply result from the effect of background genes and this might lead to misinterpretation of results.

## Results

### Infection-induced phenotypic differences between WT(Jv) and TLR9^−/−^(OR) are lost after one additional backcross generation

**Figure 1:**
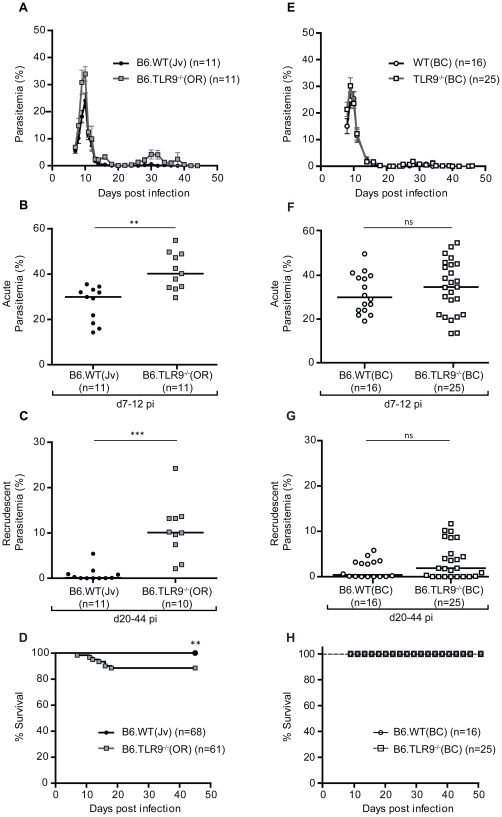
One additional backcross generation (B6.TLR9^−/−^(BC) and B6.WT(BC)) abolishes the phenotypic differences in the course of infection between B6.WT(Jv) and B6.TLR9^−/−^(OR) mice. As detailed in materials and methods, B6.WT(Jv) mice were intercrossed with B6.TLR9^−/−^(OR) mice and heterozygotes (F1) were mated to obtain matched backcrossed B6.WT(BC) and B6.TLR9^−/−^(BC) mice. The offspring were used for experiments. B6.WT(Jv) (black circles), B6.TLR9^−/−^(OR) (grey squares), B6.WT(BC) (open circles) and B6.TLR9^−/−^(BC) (open squares) mice were infected ip with 10^4^ *Pc*AS pRBCs. (**A,E**) The course of parasitemia was monitored for 44 days by microscopic examination of thin Giemsa-stained blood smears. (**B,F**) Since not all mice reached their maximum peak parasitemia on the same day, the highest parasitemia of each individual mouse between day 7 and 12 was selected as peak parasitemia and indicated as a single dot. The horizontal lines indicate the group medians. (**C,G**) Since the timing of the recrudescences varied significantly between 20 and 44 days pi, the peak parasitemia values during recrudescences between day 20 and day 44 pi are shown and represented as single dots. The horizontal lines indicate the group medians. (**D,H**) Survival was monitored until 44 days pi in the four different groups. (**A–C**) Data are representative of more than three independent experiments with at least five mice per group in each experiment. (**D**) Data are a compilation of several experiments with at least five mice per group. (**E–H**) Data are a compilation of two independent experiments with at least seven mice per group at each time point. ns, not significant. The numbers of mice (n) in each group are depicted in the graph legend. **, *p*<0.01; ***, *p*<0.001.

The infection of C57Bl/6 WT mice with *Pc*AS is characterized by an acute primary peak of parasitemia around day 9–10, followed by a chronic phase marked by one or two small recrudescences around day 30 post infection (pi). Finally, the parasite is cleared and mice survive the infection [34]. Since TLR9 is a crucial component connecting innate and adaptive immunity, it was interesting to investigate the role of TLR9 in protective immunity against blood-stage *Pc*AS. The TLR9 genotype of each mouse was confirmed by PCR (Figure S1). In first instance, we compared the course of *Pc*AS infection in the originally received B6.TLR9^−/−^(OR) mice and commercial inbred B6.WT(Jv) mice and observed a phenotype in parasitemia. Primary parasitemia peaked between 7 and 12 days pi and peak values of individual mice were significantly higher in B6.TLR9^−/−^(OR) mice compared to B6.WT(Jv) mice (Figures 1A–B; *p*<0.01). During the chronic stage of infection between day 20 and day 44 pi, B6.TLR9^−/−^(OR) mice suffered from 10-fold higher levels of recrudescent parasitemia (median = 10.1%) compared to the B6.WT(Jv) controls, which had only small recrudescences (median = 0.09%) (Figure 1C; *p*<0.001). Moreover, infected B6.TLR9^−/−^(OR) mice became considerably more anemic (Figure S2; *p*<0.001) and limited but significant mortality was observed in large cohorts among these TLR9-deficient mice, whereas B6.WT(Jv) mice were fully resistant to *Pc*AS infection (Figure 1D; *p*<0.01 by Log-rank (Mantel Cox) test). Hence, although discrepant from other published data [15], [35]–[38], the B6.TLR9^−/−^(OR) mice were less well protected against acute and chronic parasitemia.

As antibody-dependent immunity is considered essential during blood-stage malaria infection, particularly for the resolution of the chronic stage of infection [39]–[42], we tried to explain antiparasite immunity with known TLR9 effects on antibody responses [43], [44] (Figure S3). Our data suggested that IgM, IgG2b and IgG2c antibodies might play a role in the protective immunity to control acute and chronic stage *Pc*AS infection, whereas IgG1 was inversely correlated with parasite control. Serum transfer experiments were performed and significant antiparasite activity was observed only when immune sera were derived from commercial B6.WT(Jv) mice and not from B6.TLR9^−/−^(OR) mice (Figure S4). The Th_2_-skewed antibody profile in B6.TLR9^−/−^(OR) mice together with the inability of B6.TLR9^−/−^(OR) immune sera to affect primary parasitemia levels caused the impression that TLR9 liganding assists in the generation of a protective antibody profile essential for the acquisition of anti-malaria immunity. However, this was in contradiction to the results published by Seixas *et al.*, who showed that both parasitemia control and antibody production were not affected by a TLR9 deletion [35].

To determine whether the observed difference in susceptibility to *Pc*AS infection between B6.WT(Jv) and B6.TLR9^−/−^(OR) mice was due to the inactivation of the TLR9 gene or to genetic and phenotypic line differences unrelated to the targeted mutation, B6.WT(Jv) and B6.TLR9^−/−^(OR) mice were crossed and heterozygous progeny were mated to obtain matched B6.WT and B6.TLR9^−/−^ lines. These additionally backcrossed B6.TLR9^−/−^(BC) mice and their B6.WT(BC) controls were infected with 10^4^ *Pc*AS pRBCs and the course of parasitemia was monitored. However, in contrast to our observations with the B6.TLR9^−/−^(OR) mice (Figures 1A–C), no significant differences in the course of parasitemia between the B6.WT(BC) and B6.TLR9^−/−^(BC) mice were noticed (Figures 1E–G). Indeed, acute as well as recrudescent peak parasitemia values between both control and mutant lines were comparable in this experimental setup (Figures 1F–G). In addition, all B6.TLR9^−/−^(BC) mice survived the infection (Figure 1H), whereas some mortality was observed in the B6.TLR9^−/−^(OR) mice used in the initial experimental setup (Figure 1D).

These data, however, reinforce the published ones [15], [35]–[38] and called for a reconsideration of the genetic background. SNPs, the single most abundant class of genetic variation in mammals, have become the markers of choice to distinguish inbred mouse strains on a genetic basis.

**Figure 2:**
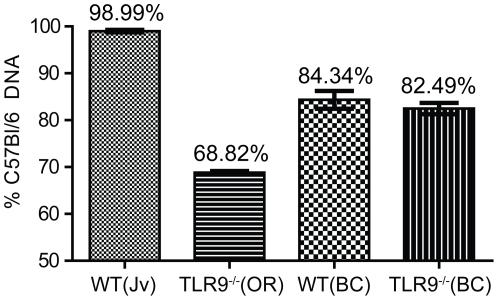
TLR9^−/−^(OR) mice are not congenic to the C57Bl/6 inbred line. The average percentage of C57Bl/6 DNA in each mouse line was determined by monitoring of the genetic background via SNP markers (Table S1). The bars represent the mean ± SEM. B6.WT(Jv) and B6.TLR9^−/−^(OR), n = 4; B6.WT(BC) and B6.TLR9^−/−^(BC), n = 3.

Extensive SNP genotyping was performed by Charles River as detailed in the Materials and Methods section. Fourteen samples were tested: B6.WT (Jv, samples 1–4), B6.TLR9^−/−^ (OR, samples 5–8), B6.WT (BC, samples 9–11) and B6.TLR9^−/−^ (BC, samples 12–14) (Table S1). In addition, the TLR9 genotype of each mouse was confirmed by PCR (Figure S1). Although we started our experiments with the certification that the B6.TLR9^−/−^(OR) mice were at least 10-fold backcrossed to an inbred C57Bl/6 background, the SNP analysis ascertained significant genetic background differences in the B6.WT(Jv) versus B6.TLR9^−/−^(OR) mice. In essence, the B6.TLR9^−/−^(OR) mice were definitely not congenic to C57Bl/6 as these animals still had large regions of 129 DNA unlinked to the *TLR9* gene. This 129 DNA originates from the ‘129’-derived embryonic stem (ES) cells carrying the targeted mutation. When considering only those SNPs that differentiate between C57Bl/6 and 129, the analysis revealed an average of 69% C57Bl/6 DNA in the B6.TLR9^−/−^(OR) mice (Figure 2; Table S1, samples 5–8). This indicates that only a maximum of two backcross generations had occurred. According to classic genetics, however, the average percentage of the genetic material after backcrossing a 129 chimera with a C57Bl/6 mouse for two generations is expected to be 75%. It might be possible that continuous long-term inbreeding of the ‘two generations backcrossed TLR9^−/−^(OR) mice’ had favored a positive selection for 129 genes, thereby increasing the percentage of 129 DNA at the expense of C57Bl/6 DNA. The additional backcross that we performed to produce matched control and mutant lines increased the degree of C57Bl/6 DNA in the new B6.TLR9^−/−^(BC) mice by approximately 15% (Figure 2; Table S1, samples 12–14), but also “generated” the new B6.WT(BC) mice with a similar level of non C57Bl/6 DNA including 129 DNA (Figure 2; Table S1, samples 9–11).

## Discussion

Genetic components determining host susceptibility to malaria are complex, both in human and in mice [45]. Considerable efforts have been made to identify the genes responsible for the development of the disease [13]. In human malaria, for example, control of parasitemia levels was found to be linked to a locus on chromosome 5q31-q33 [46].

Approximately 99% of human genes have mouse homologs and the gene order is highly conserved. Hence, the mouse has served as an appropriate experimental model that allowed to dissect complex genetic traits and to decipher immunopathological pathways of several complex human diseases. Inbred mouse strains are generated by at least 20 sequential generations of sibling mating, which drives heterozygous loci to homozygosity and increases the genetic uniformity of the line. After 20 generations of inbreeding, on average 98.6% of the genome should be homozygous. This enables researchers to conduct reproducible experiments [47]. Several studies, however, highlight the importance of the genetic background of the mouse strain used to mimic and investigate human disease phenotypes. This is illustrated by inbred mouse models of malaria infection, in which the outcome is dependent upon the genetic background of the host. Indeed, C57Bl/6 mice are resistant to infection with *Pc*AS, whereas A/J mice succumb to infection due to uncontrolled parasitemia and severe anemia accompanied by suppressed erythropoiesis [40], [48]–[50]. Quantitative trait locus (QTL) mapping studies in recombinant congenic mouse strains have identified several *Chabaudi* resistance (*Char*) loci on various chromosomes important in controlling susceptibility to *Pc*AS infection, and hence, underline the complexity and multigenicity in parasite control [51]–[54].

In the last decade, the use of mice carrying engineered genetic modifications has revolutionized the study of gene function. However, these new techniques also have their drawbacks and special care should be taken when the target genes are involved in a complex network of interacting genes and biochemical processes, which is the case for most immunological events. Indeed, the phenotype of a mutation is often tuned by a particular genomic context in which flanking genes as well as the genetic background contribute to the induced mutation (reviewed by Sigmund *et al.* [19]). As an example, outside of malaria research, an epidermal growth factor null mutation on a CF-1 background results in early embryonic death, whereas homozygous mutants on a 129/Sv background die at mid gestation. On a CD-1 background, the mutants survive for 3 weeks [20]. Hence, genes should not be studied alone but have to be seen in an epistatic multigenic network. It is extremely important to use congenic strains when evaluating phenotypes resulting from gene manipulation [19]. Congenic strains are defined as two inbred strains which are genetically identical, except for the targeted gene and its flanking region [55], [56]. Eliminating the confounding effects of background genes will improve the understanding of gene function. Most commonly used ES cell lines for gene targeting are derived from strain 129. Using traditional backcrossing methods, the 129 genome is progressively diluted. At least 7 generations of backcrossing to a maintenance inbred strain (usually C57Bl/6) are required to establish congenic lines with more than 99% genetic homogeneity. However, the region flanking the mutated gene still contains 129 DNA. It is recommended to characterize knockout phenotypes on several different and clearly defined backgrounds. More ideally, besides the conditional knockout models, the use of co-isogenic lines, defined as two inbred strains that carry different alleles of only one gene but which are otherwise genetically identical, will overcome the flanking gene problem (use of ES cells from desired strain) [19], [57]–[61].

An additional hurdle is that, although inbred strains are forced to homozygosity and genetic uniformity, minor variations within an inbred strain have been found. Moreover, when inbred strain colonies are separated and raised in a different environment or at another laboratory, substantial substrain differences may occur. The use of different substrains from the same inbred strain can hamper the reproducibility and interpretation of experimental results. Genetic variation arises by accidental genetic contamination due to errors in animal management, or by genetic drift. Genetic drift favors fixation of new mutations or the elimination of residual heterozygosity and leads to changes in inbred strains over time or between inbred lines that were physically separated (i.e. substrains). Commercial breeders have specific breeding programs to slow down genetic drift, e.g. the Genetic Stability Program (GSP) of The Jackson Laboratory [62]–[64]. It is essential to acknowledge the phenomena of spontaneous mutations and genetic drift regarding the use of WT mice from a commercial breeder or other sources as ‘controls’ when designing studies with genetically engineered mice. A solution may be to intercross both inbred WT control mice with KO mice and use enough WT and KO progeny from F2 for experimental setup. Applying this strategy will scramble the genetic background of both mouse lines and even out a large part of small line variations.

The data presented in this study highlight the importance of a well-defined homogenous genetic background - which should be as similar as possible between the control and the mutant line - in the analysis of the effect of a *TLR9* deletion on the parasitemia course during a *Pc*AS infection. In our first experimental setup, an increased susceptibility against *Pc*AS infection was observed in mice lacking TLR9, which were originally received as being at least 10 times backcrossed to the inbred C57Bl/6 strain. However, after backcrossing B6.TLR9^−/−^(OR) mice into B6.WT(Jv) for one additional generation, the TLR9 KO-associated phenotype was lost. Reassessment of the genetic identity of the original B6.TLR9^−/−^(OR) mice by analyzing 384 mouse SNP markers revealed a maximum of two backcross generations resulting in a heterogeneous genetic background composed of C57Bl/6 DNA together with large regions of 129 DNA unlinked to the TLR9 gene. Some of the B6.TLR9^−/−^(BC) mice, however, still displayed high recrudescent parasitemia values comparable to those observed in the insufficiently backcrossed B6.TLR9^−/−^(OR) mice used in the initial setup. A possible explanation is that, due to random genetic recombination in meiosis, e.g. by independent assortment of chromosomes and chromosomal crossovers, some of the new B6.TLR9^−/−^(BC) mice, which were still not congenic after an extra backcross, might still contain the 129 genes that contributed to the increased susceptibility for *Pc*AS infection. Indeed, genetic analyses of hybrids of various inbred mouse strains with different susceptibilities to *P. chabaudi* have revealed that several loci on several chromosomes are associated with susceptibility/resistance to high parasitemia (reviewed in [49]). A similar hypothesis can also be applied to the observed increased recrudescent parasitemia values observed in some of the new B6.WT(BC) mice. Indeed, these B6.WT(BC) mice have gained some 129 DNA at the expense of C57Bl/6 genetic material. Rather than being induced simply by the *TLR9* null mutation, the observed increased susceptibility phenotype might be due primarily to ‘contaminating’ 129 DNA. This theory is indeed supported by the fact that the 129 mouse strain is also susceptible for *Pc*AS infection [49]. A third possibility might be that the enhanced susceptibility phenotype is induced by epistatic interactions of uncharacterized background genes, originating from DNA of 129 and C57Bl/6 mouse strains. An illustration of a phenotype induced by the combined effect of both 129 and C57Bl/6 genetic material is provided by Bygrave *et al.* [65]. These authors have shown that, in the absence of any gene-targeted mutations, the interplay between loci of 129 and C57Bl/6 mice was sufficient to cause humoral autoimmunity. Hence, the additional intercross between B6.WT(Jv) and B6.TLR9^−/−^(OR) mice has shuffled their genetic background and evened out the phenotypic line differences which were probably due to 129 DNA and unrelated to the *TLR9* deletion. In accordance herewith, Seixas *et al.* did also not observe any effect of TLR9 deficiency on the course of parasitemia and antibody profiles during a *Pc*AS infection [35].

Guided by the results presented in this study and in addition to several other reports that stress complications in the experimental setup of knockout studies [59]–[61], it has to be acknowledged that the poor description of the mouse genetic constitution in many published reports is in sharp contradiction with the rules of classical genetics. Table 1 lists the experimental setup and results of 5 studies on the effect of a TLR9 deletion in a *Pc*AS infection. In addition, 13 studies on the role of TLR9 in other malaria models are included for further comparison. From this, it is clear that often insufficient information on the genetic setup of the used KO and control WT mice is provided. This is in part due to the free circulation of many KO mouse strains between many laboratories worldwide, sometimes resulting in the loss of the correct information on the genetic background. In addition, the WT mice are often obtained from a commercial breeder, which raises concerns about the use of these mice as controls. Indeed, even if the KO strain is sufficiently backcrossed into an inbred line, it is important to use non-transgenic control mice derived from the same inbred line, as this will minimize strain differences due to genetic drift. Alternatively, intercrossing of the control WT mice and the KO mice is convincing as a final confirmation of the obtained results, if *in vivo* neutralization of the target protein is practically unachievable. More advanced approaches to study gene function are the ‘conditional knockout’ models or the use of ES cells from the required strain [59]. The reason behind the discrepant results summarized in Table 1 concerning the role of TLR9 in malaria pathogenesis might be due to the distinct experimental designs of the mouse studies by different research groups. This problem of genetic background effects may not be limited to the role of TLR9 in *Pc*AS infections, but may also be relevant for other studies with other mouse models, even not related to malaria. Furthermore, besides host genetics, other factors may be invoked in determining susceptibility to *Pc*AS and other malaria strains, i.e. parasite substrains and genetics, breeding and housing conditions, intestinal flora, food, sex, age and also the source and route of infection [25], [66]–[69].

**Table 1:** Overview of the genetic background of TLR9^−/−^ mice in experimental malaria infections.

CONTROL STRAIN		KNOCKOUT STRAIN		TLR9 knockout phenotype	Ref.
Source	Breeding information	Source	N° of backcross generations to the WT background		
***Plasmodium chabaudi*** ** AS**					
B6.WT(?) + *Pc*AS	undefined	B6.TLR9^−/−^(?)+*Pc*AS	undefined	no difference in parasitemia, in body weight and in temperature, ↓ production of IFN-γ by splenocytes on day 8 pi	[36]
B6.WT (local breeding facility) + *Pc*AS	undefined (*pure local C57Bl/6 line)	B6.TLR9^−/−^ (Prof. Akira,Japan)^a^+*Pc*AS	undefined (*at least 8–9× on local inbred line)	no difference parasitemia, no difference in the alterations in splenic microarchitecture	[37]
B6.WT(JL) (DCs) + *Pc*AS	according to commercial breeder strategy [75]	B6.TLR9^−/−^ (Prof. Akira,Japan)^a^ (DCs)+*Pc*AS	at least 8×	no difference in parasitemia, ↓ TLR upregulation, ↓ production of IFN-γ/IL-12 by splenocytes/ DCs, ↑ resistance against LPS-induced mortality	[15]
B6.WT (?, *local breeding facility) (pDCs) + *Pc*AS	undefined (*pure local C57Bl/6 line)	B6.TLR9^−/−^ (Prof. Akira,Japan)^a^ (pDCs)+*Pc*AS	10×	↓ IFN-γ production in co-culture of pDC+iRBC, ↓ IFN-γ mRNA in pDC on day 3 pi, no difference in parasitemia, body weight and temperature	[38]
B6.WT (local breeding facility) (DCs) + *Pc*AS or *Pb*ANKA	undefined	B6.TLR9^−/−^ (Prof. Akira,Japan)^a^ (DCs)+*Pb*ANKA or *Pc*AS	undefined	↓ DC activation by parasites, no nuclear translocation of NF-κB in DCs, no difference in parasitemia (*Pc*AS), ↓ *in vivo* activation of splenic DCs, ↑ IgM on day 10 pi (*Pc*AS)	[35]
***Plasmodium berghei*** ** ANKA**					
B6.WT(?) + *Pb*ANKA	undefined	B6.TLR9^−/−^ (Prof. Akira,Japan)^a^+*Pb*ANKA	at least 10×	↓ macrophage response to pRBCs, no difference in parasitemia, in lung and hepatic pathology, in CM development and in survival	[23]
B6.WT(JL) + *Pb*ANKA	according to commercial breeder strategy [75]	B6.TLR9^−/−^(?)+*Pb*ANKA	at least 7×	no difference in parasitemia, ↑ survival, ↓ CM	[18]
B6.WT (CLEA, Japan) + *Pb*ANKA	according to commercial breeder strategy [76]	B6.TLR9^−/−^ (Prof. Akira,Japan)^a^ + *Pb*ANKA	at least 8×	no difference in parasitemia, ↑ survival, ↓CM	[22]
B6.WT (?, *local breeding facility) + *Pb*ANKA	undefined (*pure C57Bl/6 from local breeding facility)	TLR2/4/9^−/−^(Kirschning,Munich)^b^ + *Pb*ANKA	mixed (129SVxC57Bl/6)	no difference in parasitemia, CM development and survival	[24]
***Plasmodium yoelii***					
B6.WT (Kyudo,Japan) + *P. yoelii*	undefined	B6.TLR9^−/−^ (Prof. Akira,Japan)^a^ + *P. yoelii*	at least 15×	partial resistance to lethal infection (parasitemia, survival), ↓ activation of Tregs by DCs, ↑ activation of CD4^+^ T cells	[68]
Balb/c.WT (CLEA Japan) + immunization with baculovirus-based PyMSP1_19_ + *P. yoelii* 17XL	according to commercial breeder strategy [76]	Balb/c.TLR9^−/−^ (Prof. Akira,Japan) + immunization with baculovirus-based PyMSP1_19_ + *P. yoelii* 17XL	undefined	vaccine induced protection abolished, ↑ Th_2_ immune responses	[77]
***Parasite components***					
B6.WT (CLEA Japan): DCs + schizont extracts	according to commercial breeder strategy [76]	B6.TLR9^−/−^ (Prof. Akira,Japan)^a^: DCs+schizont extracts	8×	↓ schizont-induced DC activation	[27]
B6.WT (?, *CLEA Japan) (DCs) + n/sHz	undefined (*according to commercial breeder strategy [76])	B6.TLR9^−/−^ (Prof. Akira,Japan)^a^ (DCs) + n/sHz	undefined (*at least 8×)	↓ innate immune activation by n/sHz	[28]
B6.WT(JL): DCs + n/sHz	according to commercial breeder strategy [75]	B6.TLR9^−/−^ (Prof. Akira,Japan)^a^: DCs + n/sHz	more than 10× (genetic background analyzed by microsatellite analysis (Charles River Laboratories))	↓ innate immune activation by plasmodial DNA on Hz	[31]
B6.WT(JL) (macrophages) + sHz	according to commercial breeder strategy [75]	B6.TLR9^−/−^ (Prof. Akira,Japan)^a^ (macrophages) + sHz	9×	no difference in inflammatory response to sHz	[78]
B6.WT (?, *CLEA Japan) + *Pf* crude extract or + sHz	undefined (*according to commercial breeder strategy [76])	B6.TLR9^−/−^ (Prof. Akira,Japan)^a^ + *Pf* crude extract or + sHz	undefined (*at least 8×)	↓ adaptive immune responses after *Pf* crude extract immunization, no difference in potent adjuvanticity of sHz	[30]
B6.WT(JL): DCs + parasite components	according to commercial breeder strategy [75]	B6.TLR9^−/−^ (Prof. Akira,Japan)^a^: DCs + parasite components	undefined	↓ activation of DCs by MZs and iRBCs	[79]
B6.WT (?, *JL): DCs + polynucleosomes	undefined (*according to commercial breeder strategy [75])	B6.TLR9^−/−^( Prof. Akira,Japan): DCs + polynucleosomes	undefined (*several x)	little or no activation of DCs by polynucleosomes	[32]

a Generated by Hemmi *et al.* [70].

b Generated by Yasuda *et al.* [80].

* Personal communication.

↑, increased; ↓, decreased; B6, C57Bl/6; DCs, dendritic cells; CM, cerebral malaria; (p)DCs, (plasmacytoid) dendritic cells; (n/s)Hz, (natural/synthetic) hemozoin; iRBCs, infected red blood cells; JL, Jackson Laboratory; MZs, merozoites; NF-κB, nuclear factor κ-light-chain-enhancer of activated B cells; *Pb*ANKA, *Plasmodium berghei* ANKA; *Pc*AS, *Plasmodium chabaudi chabaudi* AS; *Pf, Plasmodium falciparum*; PyMSP1_19_, *Plasmodium yoelii* 19 kDa carboxyl terminus of merozoite surface protein 1; TLR9, toll-like receptor 9; Tregs, regulatory T cells.

As a conclusion, in phenotype studies of genetically modified animals, a significant impact on the phenotype may come from the culmination of small effects by many genes that may be different between wild type and modified animals. These differences may be minimized by a sufficient number of backcrosses. Ideally, gene function has to be studied between congenic or even co-isogenic strains. For the sake of reproducibility, all reports concerning genetic experiments must include detailed information on the origin and the genetic background of the studied animals.

## Materials and Methods

(Additional information on the experimental procedures is provided in supporting Text S1)

### Ethical statement

All animal experiments were conducted with the approval of the Institutional Ethics Committee under license LA121251 (Belgium) for animal welfare.

### Mice and experimental infections

Male and female C57Bl/6 wild type (B6.WT) mice were purchased from Janvier (Jv, Heverlee, Belgium). B6.TLR9 knockout mice (B6.TLR9^−/−^) were originally generated as described previously [70] in the laboratory of Prof. S. Akira (Department of Host Defense, Research Institute for Microbial Diseases, Osaka University, Osaka, Japan). They were obtained from Prof. S. Magez [Laboratory for Cellular and Molecular Immunology, Free University of Brussels (VUB), Belgium]. After receiving B6.TLR9^−/−^ breeding pairs, these mice were bred and maintained in the conventional animal facility St. Rafaël of the Rega Institute for Medical Research (University of Leuven, Leuven, Belgium). From each individual mouse, the TLR9 genotype was confirmed by PCR. B6.WT and B6.TLR9^−/−^ mice, 8–12 weeks old, were age- and sex-matched in all experiments. These mice are denoted as B6.WT(Jv) and B6.TLR9^−/−^(OR) mice.

To generate non-transgenic littermates as B6.WT controls, an intercross breeding strategy was performed. B6.WT(Jv) mice were coupled with B6.TLR9^−/−^(OR) mice to yield F1 heterozygotes. The latter were inter-mated to obtain additionally backcrossed TLR9^−/−^mice and matched WT controls in the F2 generation. For each individual mouse, the genotype was confirmed by PCR. To obtain a reasonable number of mice required for experimental setups, breeding pairs of B6.WT and B6.TLR9^−/−^ mice from the F2 generation were assembled. Henceforth, these additionally backcrossed B6.TLR9^−/−^ mice and their matched B6.WT controls were marked as B6.TLR9^−/−^(BC) and B6.WT(BC) mice. Mice were infected intraperitoneally (ip) with blood containing 10^4^ *Plasmodium chabaudi chabaudi* clone AS (*Pc*AS) parasitized red blood cells (pRBCs) (a kind gift of the late Prof. Dr. D. Walliker, University of Edinburgh, Scotland, U.K.). 10^4^ pRBCs as infectious dose was previously used by our laboratory and also by others [71]–[74]. Mice received high energy food and drinking water was supplied with para-amino benzoic acid (PABA) to improve *in vivo* parasite growth. Parasitemia of individual mice was monitored microscopically on Giemsa-stained blood smears.

### Background strain characterization testing via Single Nucleotide Polymorphisms (SNP) markers

Background strain characterization was performed at Charles River (Charles River Genetic Testing Services, Troy, NY, USA). Mouse tissue samples were tested with a background strain characterization panel to evaluate strain line purity. The panel consisted of 384 SNP markers. The SNP markers were spread across the 19 mouse autosomes and the X chromosome at about 7 Mbp intervals and were polymorphic between various strains of mice. In particular, 199 SNPs were polymorphic between C57Bl/6 and 129. An array of inbred and hybrid controls were also analyzed along with the samples. From 4 B6.WT(Jv), 4 B6.TLR9^−/−^(OR), 3 B6.WT(BC) and 3 B6.TLR9^−/−^(BC) mice, tail DNA was prepared using the Nucleospin Tissue Kit (Clontech Laboratories, Mountain View, CA, USA) on a Coulter Biomek FX robotic system (Beckman Coulter, Brea, CA, USA). After verifying yield on a 1% agarose gel or a NanoDrop Spectrophotometer, the DNA was diluted to 20–40 ng/µl with distilled H_2_O (DNase and RNase free, Gibco, Invitrogen, Carlsbad, CA, USA). Subsequently, the DNA was loaded into OpenArray chips (Applied Biosystems, Foster City, CA, USA) containing the Taqman primers and probes for the 384 SNP markers. The two possible allele-specific probes were labeled with reporter dyes VIC or FAM and contained a nonfluorescent quencher.

Each reaction was performed in a volume of 33 nanoliters in a separate through-hole on the array. The array containing the Taqman SNP assays was cycled in a Hybaid thermal cycler as follows: 1 cycle of 10 minutes at 91°C; 49 cycles of 23 seconds at 51°C, 30 seconds at 53.5°C, 13 seconds at 54.5°C, 22 seconds at 97°C and 7 seconds at 92°C; 1 cycle of 5 minutes at 20°C.

Once the array had been cycled, VIC and FAM fluorescence (corresponding to the two possible SNP alleles) were quantified by analyzing the array in an OpenArray Imager. Fluorescence data were analyzed using the OpenArray SNP Genotyping Analysis software (Applied Biosystems). Genotyping data were exported to Microsoft Excel, sorted to place the markers in chromosomal order, and pasted into a strain-specific scoring template to convert the alleles present in each sample to numerical values. Markers homozygous for the expected allele in the background to be quantified were scored as “1”. Markers heterozygous for the expected allele and another allele were scored as “0.5”. Markers homozygous for another allele were scored as “0”. The score for each marker was averaged to calculate the percent of the strain of interest in the background.

### Statistical analysis

Data are presented as scatter dot plots with the line indicating the median. Using the GraphPad Prism 5 software (GraphPad Software, San Diego, CA, USA), the nonparametric Mann-Whitney U-test was applied to determine the statistical significance of the observed differences between two groups. Survival was analyzed by the Log-rank (Mantel Cox) Test. Differences were considered statistically significant when *p*<0.05 (two-tailed).

## Supporting Information

Text S1 **Online supporting results and supporting materials and methods.** Discussion on the results of figure S3 (antibody levels) and figure S4 (antibody transfer experiment). Detailed information on the supporting materials and methods.
(XLSX)

Figure S1 **Genotype confirmation by PCR followed by DNA separation and visualization on an agarose FlashGel DNA cassette.** Genomic DNA was extracted from mouse tail samples as detailed in the supporting Text S1. The TLR9 knockout (upper gel) versus WT (lower gel) genotype was detected by PCR and the amplified products were separated and visualized on a 1.2% agarose FlashGel DNA cassette. A FlashGel DNA marker was also loaded on the gel. Both WT and TLR9^−/−^ bands have a length of approximately 340 bp.
(TIF)

Figure S2 **B6.TLR9^−/−^(OR) mice suffer from significantly more anemia than their B6.WT(Jv) counterparts during** ***Pc*** **AS infection.** Between day 8 and day 16 pi, 10 µl of blood was taken from the tail vein and with the use of the ‘SDS-haemichome method’, the concentration of hemoglobin was measured colorimetrically in B6.WT(Jv, black circles) and B6.TLR9^−/−^(OR, grey squares) mice. From each individual mouse, values of the lowest hemoglobin concentration after primary peak parasitemia are presented. The dashed line represents the hemoglobin level in naive mice, which is approximately 14 g/dl. The hemoglobin levels were restored to normal both in B6.WT(Jv) and B6.TLR9^−/−^(OR) mice around day 16 pi (data not shown). The horizontal lines indicate the group medians. Data are representative for 3 independent experiments with at least 5 mice per group for each experiment. The numbers of mice (n) in each group are depicted in the graph legend. **, *p*<0.01; ***, *p*<0.001.
(TIF)

Figure S3 **Altered IgM and IgG subtype levels after** ***Pc*** **AS infection in B6.TLR9^−/−^(OR)** ***vs.*** **B6.WT(Jv) mice.** B6.WT(Jv, black circles) and B6.TLR9^−/−^(OR, grey squares) mice were infected ip with 10^4^ *Pc*AS pRBCs and heparinized plasma samples were collected at 0, 21 and 42 days pi. The levels of IgM (**A**), IgG (**B**) and IgG subclasses IgG2c (**C**), IgG2b (**D**) and IgG1 (**E**) in naive and infected B6.WT(Jv) and B6.TLR9^−/−^(OR) were determined by ELISA and compared to a laboratory standard as detailed in the supporting Text S1. Horizontal lines indicate the group medians and each dot represents data from a single animal. Data are a compilation of two independent experiments with at least five mice per infected group at each time point. The numbers of mice (n) in each group are depicted in the graph legend. *, *p*<0.05; **, *p*<0.01; ***, *p*<0.001.
(TIF)

Figure S4 **Transfer of immune serum from B6.TLR9^−/−^(OR) mice did not affect the course of parasitemia in B6.WT(Jv) mice.** Naive B6.WT(Jv) mice were either sham-treated (black circles) or treated ip with 200 µl immune serum (d21 pi) from B6.WT(Jv) (open circles) or B6.TLR9^−/−^(OR) (grey circles) mice followed immediately by infection with 10^4^ *Pc*AS pRBCs. The course of parasitemia was monitored for 44 days by microscopic examination of Giemsa-stained thin blood smears. The horizontal lines indicate the group medians and each dot represents data from a single animal. Data are a compilation of two independent experiments with at least five mice per group at each time point. The numbers of mice (n) in each group are depicted in the graph legend. ***, *p*<0.001.
(TIF)

Table S1 **Background strain characterization via SNP markers.** The genetic background of the mice was determined by analysis of 384 SNPs. The results for 4 B6.WT(Jv), 4 B6.TLR9^−/−^(OR), 3 B6.WT(BC) and 3 B6.TLR9^−/−^(BC) are compared with the profiles of one pure C57Bl/6 and one pure 129 mouse. The location of the TLR9 gene on chromosome 9 (106,125 Kbp–106,132 Kbp) is shaded in grey. All loci that do not discriminate between C57Bl/6 and 129 are excluded (shaded in blue). Vs and Fs are the allele names (refers to VIC and FAM respectively). 1 = homozygous C57Bl/6; 0.5 = heterozygous for C57Bl/6 allele (green); 0 = homozygous for non C57Bl/6 allele (yellow). Percentage of C57Bl/6 DNA and average % C57Bl/6 DNA in each group is indicated at the bottom of the sheet.
(DOC)
