# Supplementary figures and images for: Insufficiently Defined Genetic Background Confounds Phenotypes in Transgenic Studies As Exemplified by Malaria Infection in Tlr9 Knockout Mice
Source: PLoS One. 2011 Nov 11;6(11):e27131. doi: 10.1371/journal.pone.0027131 (PMC3214040; doi:10.1371/journal.pone.0027131)

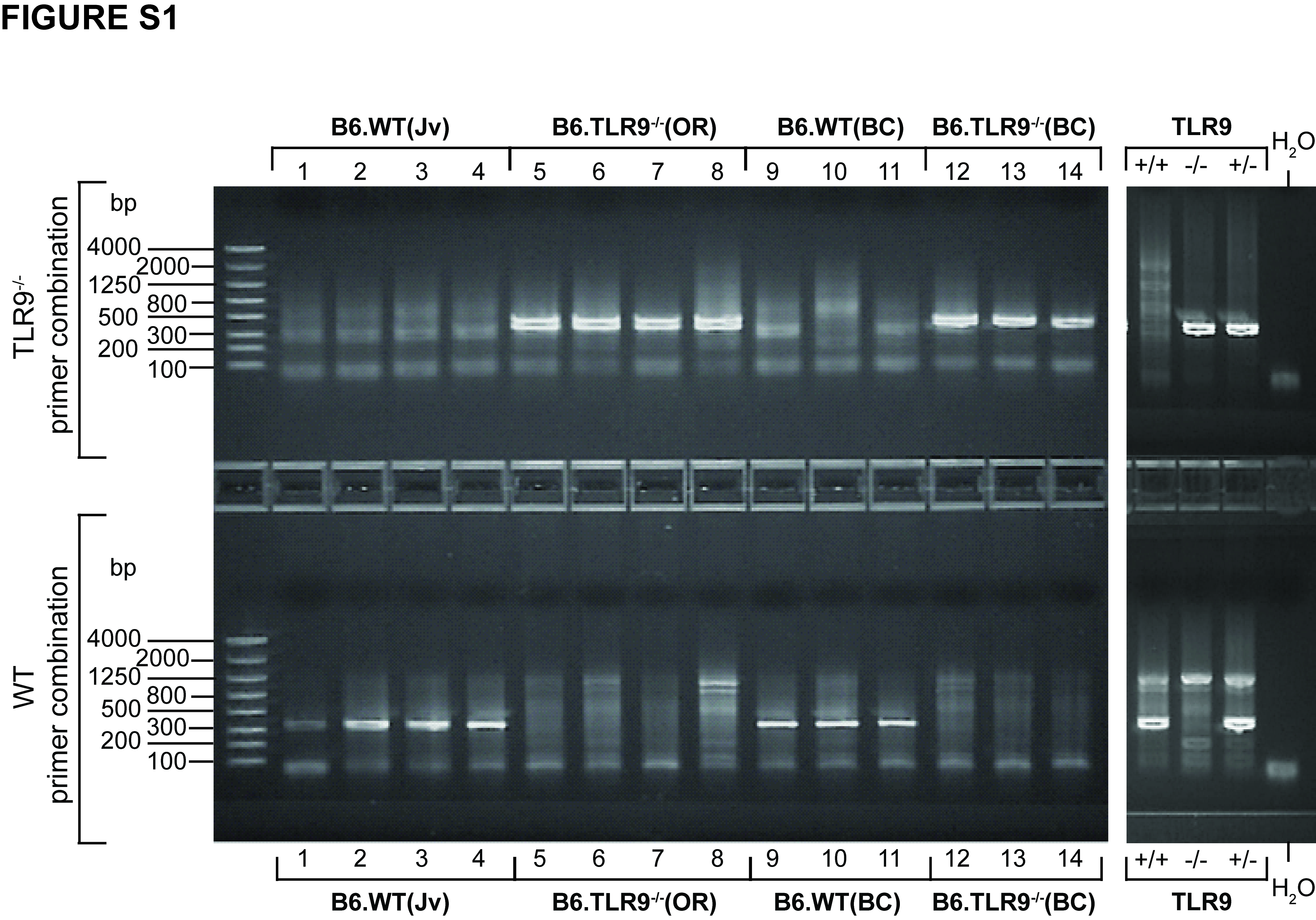

Supplement: Figure S1 — Genotype confirmation by PCR followed by DNA separation and visualization on an agarose FlashGel DNA cassette. Genomic DNA was extracted from mouse tail samples as detailed in the supporting Text S1. The TLR9 knockout (upper gel) versus WT (lower gel) genotype was detected by PCR and the amplified products were separated and visualized on a 1.2% agarose FlashGel DNA cassette. A FlashGel DNA marker was also loaded on the gel. Both WT and TLR9−/− bands have a length of approximately 340 bp. (TIF) [file pone.0027131.s002.tif]

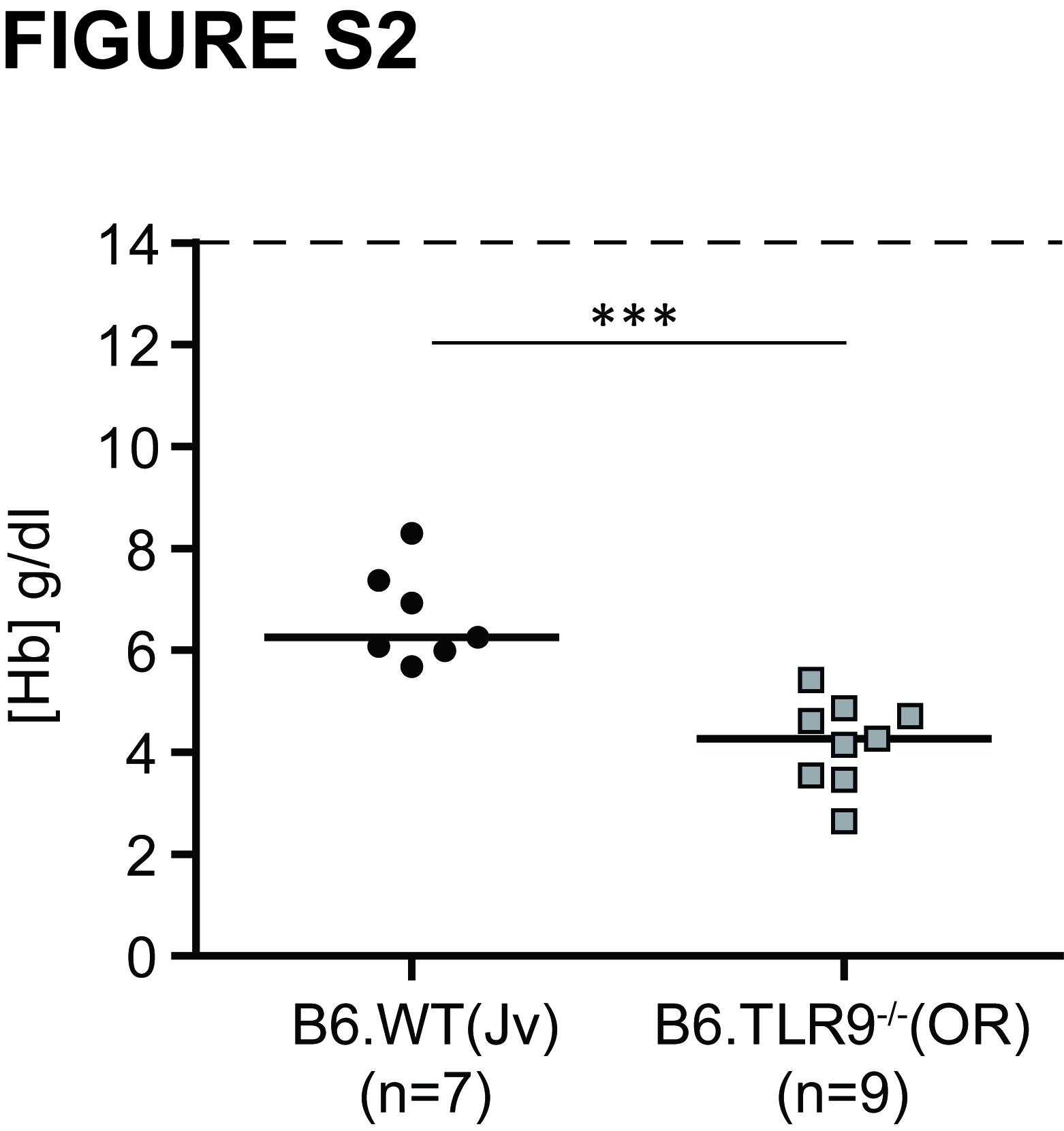

Supplement: Figure S2 — B6.TLR9−/−(OR) mice suffer from significantly more anemia than their B6.WT(Jv) counterparts during Pc AS infection. Between day 8 and day 16 pi, 10 µl of blood was taken from the tail vein and with the use of the ‘SDS-haemichome method’, the concentration of hemoglobin was measured colorimetrically in B6.WT(Jv, black circles) and B6.TLR9−/−(OR, grey squares) mice. From each individual mouse, values of the lowest hemoglobin concentration after primary peak parasitemia are presented. The dashed line represents the hemoglobin level in naive mice, which is approximately 14 g/dl. The hemoglobin levels were restored to normal both in B6.WT(Jv) and B6.TLR9−/−(OR) mice around day 16 pi (data not shown). The horizontal lines indicate the group medians. Data are representative for 3 independent experiments with at least 5 mice per group for each experiment. The numbers of mice (n) in each group are depicted in the graph legend. **, p<0.01; ***, p<0.001. (TIF) [file pone.0027131.s003.tif]

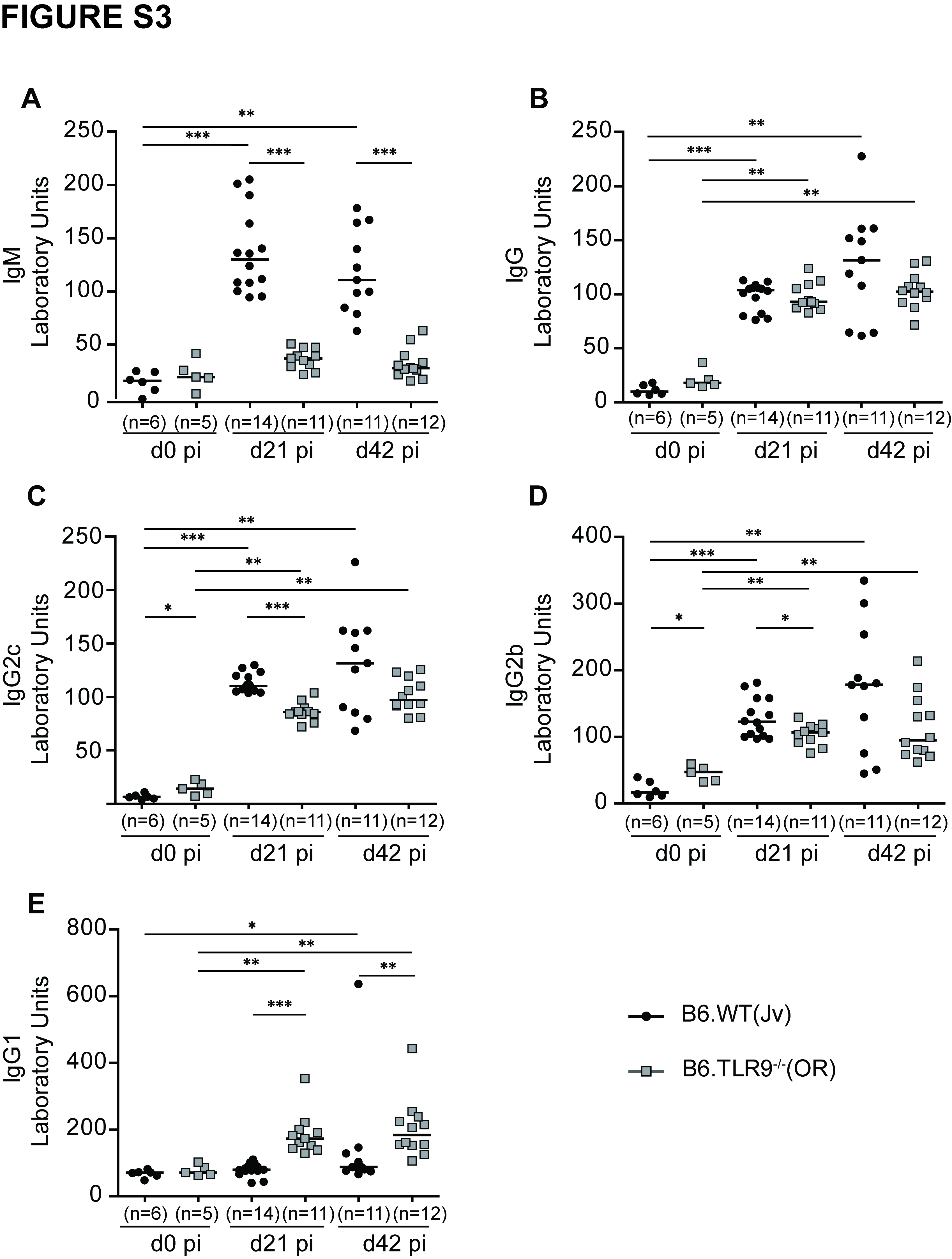

Supplement: Figure S3 — Altered IgM and IgG subtype levels after Pc AS infection in B6.TLR9−/−(OR) vs. B6.WT(Jv) mice. B6.WT(Jv, black circles) and B6.TLR9−/−(OR, grey squares) mice were infected ip with 104 PcAS pRBCs and heparinized plasma samples were collected at 0, 21 and 42 days pi. The levels of IgM (A), IgG (B) and IgG subclasses IgG2c (C), IgG2b (D) and IgG1 (E) in naive and infected B6.WT(Jv) and B6.TLR9−/−(OR) were determined by ELISA and compared to a laboratory standard as detailed in the supporting Text S1. Horizontal lines indicate the group medians and each dot represents data from a single animal. Data are a compilation of two independent experiments with at least five mice per infected group at each time point. The numbers of mice (n) in each group are depicted in the graph legend. *, p<0.05; **, p<0.01; ***, p<0.001. (TIF) [file pone.0027131.s004.tif]

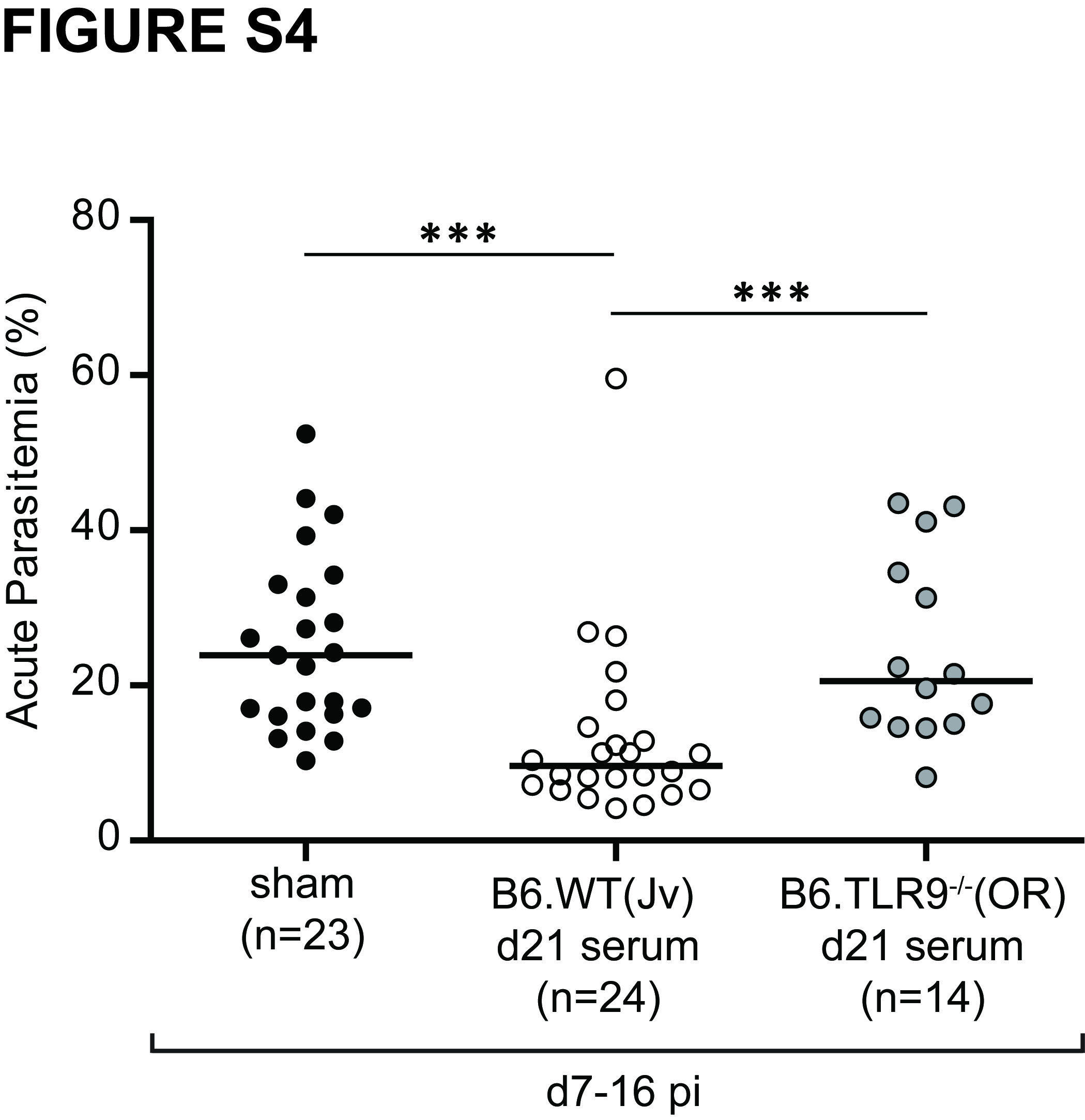

Supplement: Figure S4 — Transfer of immune serum from B6.TLR9−/−(OR) mice did not affect the course of parasitemia in B6.WT(Jv) mice. Naive B6.WT(Jv) mice were either sham-treated (black circles) or treated ip with 200 µl immune serum (d21 pi) from B6.WT(Jv) (open circles) or B6.TLR9−/−(OR) (grey circles) mice followed immediately by infection with 104 PcAS pRBCs. The course of parasitemia was monitored for 44 days by microscopic examination of Giemsa-stained thin blood smears. The horizontal lines indicate the group medians and each dot represents data from a single animal. Data are a compilation of two independent experiments with at least five mice per group at each time point. The numbers of mice (n) in each group are depicted in the graph legend. ***, p<0.001. (TIF) [file pone.0027131.s005.tif]
